# Ethnopharmacological survey among migrants living in the Southeast Atlantic Forest of Diadema, São Paulo, Brazil

**DOI:** 10.1186/1746-4269-6-29

**Published:** 2010-10-29

**Authors:** Daniel Garcia, Marcus Vinicius Domingues, Eliana Rodrigues

**Affiliations:** 1 Department of Biology, Universidade Federal de São Paulo, Rua Arthur Ridel, 275 CEP, 09941-510, Diadema, S.P., Brazil; 2 Department of Psychobiology, Universidade Federal de São Paulo, Rua Botucatu, 862 - 1º andar - Edifício Biomédicas CEP 04023-062, São Paulo, S.P., Brazil

## Abstract

**Background:**

Understanding how people of diverse cultural backgrounds have traditionally used plants and animals as medicinal substances during displacements is one of the most important objectives of ethnopharmacological studies. An ethnopharmacological survey conducted among migrants living in the Southeast Atlantic Forest remnants (Diadema, São Paulo, Brazil) is presented herein.

**Methods:**

Ethnographical methods were used to select and interview the migrants, and botanical and zoological techniques were employed to collect the indicated resources.

**Results:**

We interviewed five migrants who described knowledge on 12 animals and 85 plants. Only 78 plants were present in Diadema, they belong to 37 taxonomic families; 68 were used exclusively for medicinal purposes, whereas 10 were reported to be toxic and/or presented some restriction of use. These taxa were grouped into 12 therapeutic categories (e.g., gastrointestinal disturbances, inflammatory processes or respiratory problems) based on the 41 individual complaints cited by the migrants. While the twelve animal species were used by the migrants to treat nine complaints; these were divided into six categories, the largest of which related to respiratory problems. None of the animal species and only 57 of the 78 plant species analysed in the present study were previously reported in the pharmacological literature; the popular knowledge concurred with academic findings for 30 of the plants. The seven plants [*Impatiens hawkeri *W. Bull., *Artemisia canphorata *Vill., *Equisetum arvensis *L., *Senna pendula *(Humb. & Bonpl. ex Willd.) H.S. Irwin & Barneby, *Zea mays *L., *Fevillea passiflora *Vell. and *Croton fuscescens *Spreng)] and the two animals (*Atta sexdens *and *Periplaneta americana*) that showed maintenance of use among migrants during their displacement in Brazilian territory, have not been studied by pharmacologists yet.

****Conclusions**:**

Thus, they should be highlighted and focused in further pharmacology and phytochemical studies, since the persistence of their uses can be indicative of bioactive potentials.

## Background

Cultural mixing mediated by the migration of people around the world has generated increasing interest in recent years within the field of ethnopharmacology [1]. Medicinal plants have been used by human societies throughout history, also across geographical barriers [2]. The continuous use of certain plants and animals for medicinal purposes over time reflects their potential therapeutic value. Such substances become even more promising when they are persistently used by migrating human groups despite the considerable distances travelled and the consequent exposure to different cultures and vegetal resources. Numerous studies have collected information on medicinal plants from ethnic groups who migrated from Mexico to the U.S.A. [3,4]; from Haiti to Cuba [5]; from Africa to South America [6]; from Africa to Brazil [7]; from Colombia to London [8]; from Suriname to the Netherlands [9]; from Albania to southern Italy [10,11]; from Germany to eastern Italy [12]; and from Europe and Africa to eastern Cuba [1,13]. However, few studies have focused on migration within a country, such as that described by Rodrigues et al. [14] regarding migrants from northeastern Brazil who currently occupy the southeast.

Brazil offers a favourable environment for studies focused on migration and medicinal plants/animals because it possesses a large area of 8,514,876.599 km^2 ^[15] and boasts high indices of cultural and biological diversity. Brazil is inhabited by rural and urban populations of 232 indigenous ethnic groups [16], 1,342 Quilombola groups (descendants of Afro-Brazilian people) [17], and mestizo groups derived from the miscegenation of Indian, Black, European and Asiatic people. Brazil also houses 55,000 species of higher plants [18] and almost 7% of global animal diversity was described (ca. 100,000 out of 1.5 million), though some estimates suggest that this number is significantly higher [19]. Migration between regions of this country encourages contact with the rich biological and cultural diversity and allows interpersonal interactions that contribute to the transformation of local medicinal therapies.

According to Simões and Lino [20], the original Atlantic Forest covered approximately 1.3 million km^2^, spanning 17 Brazilian states from south to northeast; however, it currently covers only 14 states, and its area has been reduced to 65,000 km^2^. Despite considerable fragmentation, the Atlantic Forest still contains more than 20,000 plant species (8,000 endemic) and 1,361 animal species (567 endemic). It is the richest forest in the world in wood plants per unit area; the southern Bahia, for example, holds a record of 454 different species/ha [21].

The objective of this study was to perform an ethnopharmacological survey among migrants from northeastern and southeastern Brazil who currently live in Atlantic Forest remnants in the municipality of Diadema (São Paulo state, southeastern Brazil). We attempted to understand how the medicinal use of certain plant and animal changed as a result of the migrants' contact with new therapies, diseases and natural resources found in Diadema. These findings were classified as either: maintenance, replacement, incorporation or discontinuation of plants/animals use.

These objectives are in agreement with several stated goals of ethnopharmacology, namely, to investigate how migration can influence knowledge of medicinal plants/animals, the extent to which displaced people incorporate new species into their therapeutic methods, and, in particular, why individuals sometimes persistently adhere to old customs, before or even after they are exposed to new possibilities. Therefore, we adopt the hypothesis that the use of plants/animals as medicines is influenced by migratory movements, and access to natural resources available in the municipality of Diadema.

## Methodology

### Fieldwork

**Figure 1:**
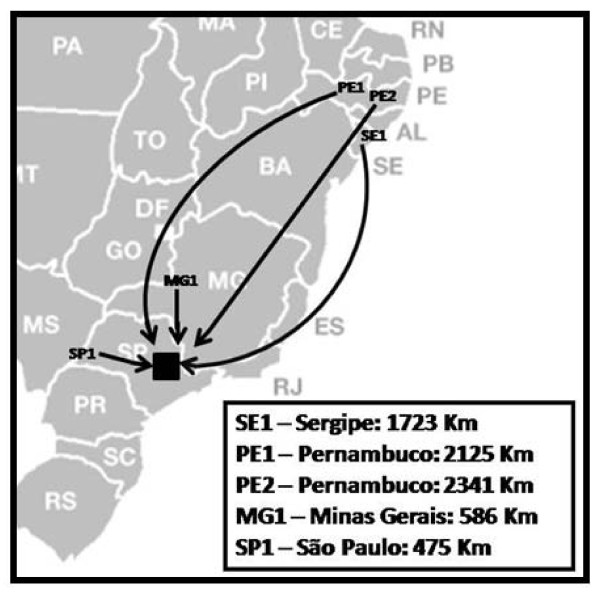
**Location of the Municipality of Diadema, in São Paulo state, southeastern Brazil (black square)**. Interviewees' migration from their cities of origin to Diadema, being PE (Pernambuco state), SE (Sergipe), MG (Minas Gerais) and SP (São Paulo), and the distance of the displacement in each case (in Km).

One of the authors (D. Garcia) spent 14 months (September 2007 to November 2008) in the municipality of Diadema, São Paulo, SP, Brazil (23°41'10"S, 46°37'22"W) (Figure 1), selecting, observing and interviewing migrants living in the Atlantic Forest remnants. Diadema is located 16 kilometres from the capital São Paulo, covers an area of 30.65 km^2^, and is occupied by 394.266 inhabitants [15], most of whom are migrants from other regions of Brazil. The municipality has a literacy rate of 6.8% [22], and its Human Development Index is 0.79 [23]. The Atlantic Forest remnants found in this city are rich in plants that are either native or introduced by the influence of those migrants present both in urban and rural areas.

Migrants who had relevant knowledge regarding the use of plants and animals for medicinal purposes were selected for interviews following the purposive sampling method [24]. Thus, we sought information about the presence of migrants from herb traders, health care professionals, and some local prayer-makers. According to Bernard [24], this sampling is also known as judgment sampling, utilized during qualitative research in particular in those populations that are difficult to be localized, the researcher selects interviewees based on their trial that they meet the criteria for the study of the phenomenon of interest. After identifying potential interviewees, the researcher visited them to determine whether they did indeed possess knowledge on medicinal plants and whether they wanted to take part in this study. The ethnopharmacological study was approved by the Ethics Committee of Universidade Federal de São Paulo (UNIFESP's Ethics Committee on Research 1969/07) and Conselho de Gestão do Patrimônio Genético (No. 02000.001 049/2008-71). The interviewees also signed consent forms granting permission to access their knowledge and collect botanical and zoological material.

Personal and ethnopharmacological data from the interviewees were obtained through informal and semi-structured interviews [24] that addressed the following topics: personal details and migration history (name, sex, age, religion, marital status, place of birth, migration, main occupation, grade of schooling) as well as ethnopharmacology (name of natural resource, use, part used, formula, route of administration, contraindications, dosages, restrictions of use). The author (D. Garcia) has visited each interviewee at least 12 times, in order to fill in the forms mentioned above, as well as to understand their processes of acquiring knowledge in depth.

Each medicinal plant was collected in the presence of the person who described it during the interviews, in accordance with the methods suggested by Lipp [25]. The plants' scientific names were determined by specialists from the Instituto de Botânica do Estado de São Paulo (IB), and vouchers were deposited at the Herbário Municipal de São Paulo (PMSP). The animals collected were placed in glass vials containing 70% ethyl alcohol, and their subsequent identification and deposit were performed by zoologists from the Museum of Zoology, Universidade de São Paulo (MZUSP) and the Bioscience Institute from Universidade de São Paulo (IB-USP).

When interviewees cited plants and animals that were used only in their cities of origin, i.e., not available in Diadema, photos from the literature and other information (e.g., popular name, habits and habitat) were used to identify them to at least the genus level. These organisms are marked with asterisks throughout the text and in Table 1. *The Herpetofauna of the Northeast Atlantic Forest *[26] and *The Herpetofauna of Caatingas and Altitudes Areas of the Brazilian Northeast *[27] were used as identification guides. For plants, we also consulted *Medicinal Plants in Brazil - Native and Exotic *[28].

**Table 1:** The 12 animals indicated by migrant PE2, their popular and scientific names, complaints (part used), formula and route of administration.

**Popular name **^dynamic of use^	Scientific name or only genus (family/class) *Voucher*	**Complaint (part used) - formula **- **route of administration**
**1- Snake (cobra)°**	*Chironius *sp., *Liophs *sp. (Colubridae/Reptilia)* or *Bothops *sp. (Viperidae/Reptilia)*	Bronchitis (skin) - powder - ingested

**2- Rattlesnake (cascavel)°**	*Crotalus *cf. *durissus *L. (Viperidae/Reptilia)*	Back pain (fat) - in natura - ingested
		Bronchitis (rattle) - tie it in the neck - topic
		Heart problems (tooth) - put it in the pocket of shirt

**3- Cururu frog (sapo-cururu)°**	*Rhinella *sp. (Bufonidae/Amphibia)*	Cancer of skin (whole animal) - in natura: tie it on the cancer for some time each day - topic

**4- Alligator (jacaré)°**	*Crocodilus *sp., *Cayman *sp. or *Paleosuchus *sp. (Alligatoridae/Reptilia)*	Apoplexy (skin) - syrup of skin powder - ingested
		Bronchitis (bone) - powder - ingested

**5- Turtle (tartaruga)°**	*Geochelone *sp. (Testudinidae/Reptilia)*	Bronchitis and asthma - (turtleshell) - powder - ingested

**6- Capybara (capivara)°**	*Hydrochoerus *cf. *hydrochaeris *L. (Hydrochaeridae/Mammalia)*	Bronchitis and asthma - (skin) - powder - ingested

**7-Iguana (iguana)°**	*Iguana *cf. *iguana *L. (Iguanidae/Reptilia) *	Osteoporosis and rheumatism (bone) - powder - ingested

**8- Ant (formiga) **^□^	*Atta sexdens *L. (Formicidae/Insecta) *Garcia 001*	Epilepsy (anthill) - in natura - ingested

**9- Cockroach (barata) **^□^	*Periplaneta americana *L. (Blattidae/Insecta) *Garcia 002*	Bronchitis and asthma (whole animal) - powder - ingested

**10- Water cockroach (barata d'água)°**	*Abedus *sp., *Belostoma *sp. or *Diplonychus *sp. (Belostomatidae/Insecta)*	Bronchitis and asthma (whole animal) - powder - ingested

**11- Lizard (calango)°**	*Placosoma *sp. (Gymnophthalmidae/Reptilia)*	Wounds in the body (skin) - powder - ingested

**12- Armadillo-ball (tatu-bola)°**	*Tolypeutes *sp. (Dasypodidae/Mammalia)*	Wounds in the body (skin) - powder - ingested

Marked by (^□^) the two animals whose use had been maintained, while 10, marked by (°) are those whose uses have fallen into disuse.

* Animals that couldn't be collected because were not available in Diadema

### Database survey

For the plants and animals identified to the species level, we searched the bibliographic databases PUBMED [29] and SCIFINDER [30] to determine whether they had been targets of previous pharmacological studies. To determine the origin of each plant species, we consulted the *Dictionary of Useful Plants: exotic and native *[31].

### Dynamics of use

During our field work, we made an effort to understand the dynamics of use for each resource and classified them into the following four categories: *maintenance of use *(resource used for the same purpose in the migrant's city of origin and in Diadema), *replacement *(resources that were replaced when migrants arrived in Diadema because the original product was not available in Diadema or was less effective than the new resource), *incorporation *(resources used for the first time in Diadema to treat diseases common to larger cities, such as hypertension, diabetes and anxiety, which were not common in their homeland), and finally *discontinued use *(resources that are no longer used in Diadema, usually because they are not available).

### Data analysis

The level of homogeneity between plant information provided by different migrants was calculated using the Informants' Consensus Factor, *Fic *[32]. This term is calculated as *Fic *= *Nur *- *Nt */(*Nur *- 1), where *Nur *is the number of use reports from informants for a particular plant-usage category and *Nt *is the number of taxa or species used for that plant usage category across all informants. Values range between 0 and 1, with 1 indicating the highest level of informant consent. For instance, if certain taxa are consistently used by informants, then a high degree of consensus is reached and medicinal traditions are viewed as well-defined [33].

## Results and Discussion

### Migrant Interviews

Despite the fact that Diadema is composed by thousands of migrants, we observed that only a few have retained traditional knowledge pertaining to medicinal plants and animals. Some considerations should be made, in order to justify our decision of conducing a qualitative approach, in depth, with the sample of interviewees obtained during the two months prior to the start of the study. During this time we observed that in many cases, this knowledge has fallen into disuse because of: a) a cultural adaptation to the new city, b) the ease of conventional medical care, c) forest degradation, which restricts use of local plants and animals, furthermore d) many migrants have shown concern to participate in the study, since in the past they suffered persecution from government agencies and physicians, who eventually restrained their medical practice.

The five selected interviewees migrated from northeast and southeast Brazil and established themselves in Diadema in the 1940 s. Three were born in the northeast: two in Pernambuco state (coded as PE1 and PE2) and one in Sergipe state (SE1). The two remaining migrants were born in the southeast: one in Minas Gerais state (MG1) and one in inland São Paulo state (SP1) (Figure 1). All interviewees were Catholic, married and retired, with the exception of PE1 and PE2 who sell medicinal plants. Their average age was approximately 68 years old (ranging from 53 to 80 years old), and their level of education was semi-illiterate to illiterate. They learned about the medicinal uses of plants and animals from their parents and grandparents (Brazilian natives, European and African descendants) in their homelands. All interviewees arrived in the city of Diadema as adults, and some had migrated through different regions of Brazil, accumulating knowledge on natural resources from human and biological sources. In Diadema, they acquired knowledge from neighbours, books, media (radio, television, magazines), and personal experiences.

### Plants: dynamics of use

The migrants described their knowledge of 85 plant specimens. As can be seen in Table 2, 78 of them were available in Diadema and were collected, resulting in 65 plant species, the remaining 13 could only be identified to the generic level. The plants belong to 37 taxonomic families, with Asteraceae (16 species), Lamiaceae (8) and Euphorbiaceae (7) as the most common. Previous studies have shown that Asteraceae species are the group most commonly reported to have potential pharmacological properties, not only in the Atlantic Forest [34-36] but also in other Brazilian biomes such as the Amazon Forest [37] the pantanal wetlands [38] and the cerrado savannahs [39]. In a review focusing on plants with possible action/effects on the central nervous system that were indicated by 26 Brazilian indigenous peoples occupying different Brazilian biomes [14], Asteraceae was the second most commonly cited family. The same pattern has been detected in other countries, such as Mexico [40]. One factor that may explain the common use of this taxonomic family is the large number of species belonging to it - about 20,000 [41]. Asteraceae also has a wide geographical distribution, both in Brazil and throughout the world [42], which facilitates its use by various cultures.

**Table 2:** The 78 plant specimens used by five Diadema's migrants (MG1, SP1, PE1, PE2, SE1)*.

Popular(s) name(s) **(migrant)**^dynamic of use^	Specimen (family) *Voucher*	Origin - geographical distribution - cultivated (C) or spontaneous (S)	Use (part)	Formula and route of administration	Pharmacological studies
1-Alamanda-amarela (SE1^□^, PE1^Δ^)	*Allamanda cathartica *L. (Apocynaceae) *Garcia 076*	Native - Brazilian territory (C)	Toxic (whole plant)	Any oral dose is dangerous	**Healing activity **[65]

2-Alecrim (MG1)^□^	*Rosmarinus officinalis *L. (Lamiaceae) *Garcia 060*	Exotic - all countries with temperate climate (C)	Muscle pain* (leaves)	Decoction - massage	**Antibacterial effects **[66], **antimicrobial effect **[67], **anti-inflammatory and anti-tumor effects **[68], **cause reduction of reproductive fertility in male rats **[69], **antinociceptive effect **[70], **mosquito repellent activity **[71], **antidiabetic and antioxidant properties **[72]

3-Alecrim-do-campo (SE1)^□^	*Baccharis dracunculifolia *DC (Asteraceae) *Garcia 021*	Native - central Brazil (S)	Soothing (aerial parts)	Smoking - inhalation	**Bactericidal activity **[73], **cytotoxic **[74], **antiulcerogenic **[75], **antimicrobial and antifungal **[76]**and anti-inflammatory **[77]

4-Alfavaca (SP1)^□^	*Ocimum selloi *Benth. (Lamiaceae) *Garcia 033*	Native - northeast to south Brazil (C)	Soothing (aerial parts)	Infusion - inhalation	**Mosquito repellent activity **[78]
				
			Bronchitis (leaves)	Syrup - ingestion	

5-Algodão (MG1)^□^	*Gossypium *sp. (Malvaceae) *Garcia 066*	No data (C)	Anti-inflammatory (leaves)	Infusion - inhalation	Not consulted

6-Algodão-do-mato (MG1, PE2)^□^	*Asclepias curassavica *L. (Apocynaceae) *Garcia 037*	Exotic - Brazilian territory (S)	Toxic* (whole plant)	Any oral dose is dangerous	**Cancer and warts treatment **[79]**and poisoning **[80]

7-Almeirão-boca-de-leão (SE1)^Δ^	*Hypochoeris *sp. (Asteraceae) *Garcia 009*	No data (S)	Liver pain (leaves)	In natura - ingestion	Not consulted

8-Amendoim-bravo, burra-leiteira (MG1, SP1, SE1, PE1, PE2)^□^	*Euphorbia heterophylla *L. (Euphorbiaceae) *Garcia 047*	Native - Americas (S)	Toxic* (whole plant)	Any oral dose is dangerous	**Cytotoxic properties **[81]

9-Anador (SE1)^□^	*Alternanthera *sp. (Amaranthaceae) *Garcia 039*	No data (C)	Soothing, headache, pain in the body (leaves)	Infusion - ingestion	Not consulted

10-Arnica (PE1)^□^	*Porophyllum ruderale *(Jacq.) Cass. (Asteraceae) *Garcia 075*	Native - Brazilian territory (S)	Muscle pain* (aerial parts)	Decoction - massage	**Anti-inflammatory **[82]

11-Aroeira (MG1)^□^	*Schinus terebinthifolius *Raddi (Anacardiaceae) *Garcia 035*	Native - northeast to south Brazil (S)	Diuretic (leaves)	Infusion - ingestion	**Antifungal activity **[83]**and antibacterial **[84]

12-Arruda (MG1, PE1, PE2)^□^	*Ruta graveolens *L. (Rutaceae) *Garcia 028*	Exotic - Brazilian territory (C)	Earache and conjunctivitis/styl* (leaves)	In natura - topic	**Antifertility **[85], **fungicide **[86], **cytotoxic **[87], **abortive **[88], **anti-tumour **[89], **anti-inflammatory **[90], **antiarrhythmic **[91]**and antimicrobial **[92]
			Muscle pain (leaves)	Decoction - massage	

13- Assa-peixe (MG1, SE1)^□^	*Vernonia *sp. (Asteraceae) *Garcia 048*	No data (S)	Bronchitis (leaves)	Infusion - ingestion	Not consulted
			Expectorant (leaves)	Infusion - inhalation	
			Healing wounds (leaves)	infusion - plaster	

14-Avelóz (PE1, PE2)^□^	*Euphorbia tirucalli *L. (Euphorbiaceae) *Garcia 046*	Exotic - Brazilian territory (C)	Toxic* (whole plant)	Restricted use (reports of blindness)	**Anti-tumour activity **[93], **cause eye injury **[94]**and effect against arthritis diseases **[95]
			Breast cancer* (latex)	Macerate - ingestion	

15-Azaléia (PE1)^Δ^	*Rhododendron simsii *Planch. (Ericaceae) *Garcia 043*	Exotic - Brazilian territory (C)	Toxic (whole plant)	Any oral dose is dangerous	**Antioxidative **[96]

16-Bálsamo (MG1, SP1, PE1, SE1)^□^	*Sedum *sp. (Crassulaceae) *Garcia 038*	No data (C)	Earache (leaves)	In natura - topic	Not consulted
			Laxative (aerial parts)	In natura - ingestion	

17-Boldo-do-Chile, figatil (PE1^□^, SE1^Δ^)	*Vernonia condensata *Baker (Asteraceae) *Garcia 001*	Exotic - northeast to southeast Brazil (C)	Liver pain* (leaves)	Infusion - ingestion	**Anti-ulcerogenic **[97]**and analgesic and anti-inflammatory **[98]

18-Brinco-de-princesa (SE1)^Δ^	*Alpinia zerumbet *(Pers.) B.L. Burtt & R.M. Sm. (Zingiberaceae) *Garcia 018*	Exotic - Brazilian territory (C)	Sedative (flowers)	Infusion - ingestion	**Antihypertensive effects **[99], **antinociceptive **[100], **anti-amoebic activity **[101]**and hepatoprotector **[102]

19-Café (MG1)^□^	*Coffea arabica *L. (Rubiaceae) *Garcia 030*	Exotic - Brazilian territory (C)	Diabetes (ripe fruits)	Infusion	**Antioxidant **[103]
			Sinusitis (powder fruit)	Infusion	

20-Cana-do-brejo (SP1, PE2)^□^	*Costus spiralis *(Jacq.) Roscoe (Costaceae) *Garcia 019*	Native - northeast and southeast Brazil (S)	Laxative and rheumatism (leaves)	Infusion or decoction - ingestion	**Antiurolithiatic **[104]

21-Cânfora (MG1, PE1, SE1)^□^	*Artemisia canphorata *Vill. (Asteraceae) *Garcia 045*	Exotic - Brazilian territory (C)	Muscle pain (whole plant)	Decoction - massage	No data found

22-Capim-limão (MG1, SE1, PE2)^□^	*Cymbopogon citratus *DC. - Stapf. (Poaceae) *Garcia 026*	Exotic - tropical countries (C)	Bronchitis* (leaves)	Syrup - ingestion	**Anxiolytic **[105], **larvicidal activity **[106], **antibacterial **[107], **antimalarial activity **[108], **insect repellent **[109], **hypoglycemic and hypolipidemic effects **[110]**and antimicrobial activity **[92]
			Sedative* (leaves)	Infusion - ingestion	

23-Capuchinha (SP1, MG1)^Δ^	*Tropaeolum majus *L. (Tropaeolaceae) *Garcia 057*	Exotic - south and southeast Brazil (C)	Ulcer and laxative (aerial parts)	Infusion or in natura - ingestion	**Antitumor activity **[111]

24-Carqueja (MG1)^□^	*Baccharis trimera *(Less) DC (Asteraceae) *Garcia 027*	Native - south and southeast Brazil (C)	Diabetes* (whole plant)	Macerate - ingestion	**Antihepatotoxic properties **[112], **anti-inflammatory and analgesic activity **[113], **relaxant effect **[114], **anti-proteolytic and anti-hemorrhagic properties **[115], **antioxidant compounds **[116], **antidiabetic activity **[117]**and for losing weight **[118]

25-Carrapicho (SE1^#^, MG1^□^)	*Acanthospermum australe *(Loefl.) Kuntze (Asteraceae) *Garcia 052*	Native - Brazilian territory (S)	Wounds in the body (roots)	Medicinal wine - ingestion	**Antimalarial activity **[119]**and antifungal activity **[120]

26-Cavalinha (MG1)^□^	*Equisetum arvensis *L. (Equisetaceae) *Garcia 051*	Exotic (C)	Diuretic (leaves)	Infusion - ingestion	No data found

27-Cipó-cruz (SE1, PE2)^Δ^	*Serjania *sp. (Sapindaceae) *Garcia 012*	No data (S)	Reduces cholesterol and diarrhea (leaves)	Macerate - ingestion	Not consulted
			External allergies, wounds in the body and detoxifying (leaves)	Infusion - bath	

28-Comigo-ninguém-pode (PE1)^□^	*Dieffenbachia *sp. (Araceae) *Garcia 071*	No data (C)	Toxic (whole plant)	Any oral dose is dangerous	Not consulted

29-Dormideira (SE1)^□^	*Mimosa pudica *L. (Fabaceae s.l.) *Garcia 069*	Exotic - Brazilian territory (C)	Healing wounds (aerial parts)	In natura - plaster	**Antidepressant activity **[121], **antitoxin of the snake *Naja kaouthia ***[122], **anticonvulsant **[123]**and for reproductive problems **[124]

30-Embaúba (MG1, SE1)^□^	*Cecropia pachystachya *Tréc. (Cecropiaceae) *Garcia 068*	Native - south to northeast Brazil (S)	Bronchitis* (powder fruit)	Syrup - ingestion	**Antioxidative activity **[125], **cardiotonic and sedative effects **[126]**and anti-inflammatory **[127]
			Toxic (sap)	Any oral dose is dangerous	

31-Erva-cidreira (MG1, SE1, PE2)^□^	*Lippia alba *(Mill.) N. E. Br. (Verbenaceae) *Garcia 005*	Native - almost all Brazilian territory (S)	Expectorant* (aerial parts)	Infusion - inhalation	**Treatment of respiratory diseases **[128], **antiulcerogenic activity **[129], **sedative and anticonvulsant effects **[130], **antiviral and antiherpes **[131]
			Sedative* (aerial parts)	Infusion or decoction - ingestion	

32-Erva-de-bicho (SE1)^□^	*Ludwigia *sp. (Onagraceae) *Garcia 078*	No data (S)	Hemorrhoid (whole plant)	Decoction - bath	Not consulted

33-Erva-doce, funcho (MG1, SP1, PE1, PE2)^□^	*Foeniculum vulgare *Mill. (Apiaceae) *Garcia 064*	Exotic -Brazilian territory (C)	Sedative (whole plant)	Infusion - ingestion	**Antimicrobial activity **[132], **anti-inflammatory, analgesic and antioxidant activities **[133], **acaricidal activity **[134], **antifungal effect **[135], **antithrombotic activity **[136]**and larvicidal activity of the mosquito *Aedes aegypti ***[137]
			Bronchitis* (whole plant)	Infusion - inhalation	
			Laxative (whole plant)	Infusion or macerate - ingestion	

34-Eucalipto, vick (MG1^□^, PE1^Δ^, PE2^Δ^, SE1^□^)	*Eucalyptus globulus *Labill. (Myrtaceae) *Garcia 055*	Exotic (C)	Sinusitis* (leaves)	Infusion - inhalation	**Antihyperglycemic actions **[138], **analgesic and anti-inflammatory effects **[139], **antimicrobial activity **[140]**and antibacterial effects **[141]

35-Fedegoso (MG1)^□^	*Senna pendula *(Humb. & Bonpl. ex Willd.) H.S. Irwin & Barneby (Fabaceae s.l.) *Garcia 034*	Native - Brazilian territory (S)	Osteoporosis prevention (roots)	Medicinal wine - ingestion	No data found

36-Feijão-guandu (SP1)^□^	*Cajanus cajan *(L.) Millsp. (Fabaceae s.l.) *Garcia 003*	Exotic - Brazilian territory (C)	Bronchitis (leaves)	Infusion - ingestion or inhalation	**Treatment of postmenopausal osteoporosis **[142], **antileishmanial and antifungal activity **[143]**and hypocholesterolemic effect **[144]

37-Folha-santa, folha-da-fortuna (MG1, SP1, PE1)^□^	*Bryophyllum pinnatum *(Lam.) Oken (Crassulaceae) *Garcia 040*	Exotic - Brazilian territory (C)	Lumbar pain* (leaves)	In natura - plaster	**Antibacterial activity **[145], **anti-ulcer **[146], **antimicrobial **[147], **antinociceptive, anti-inflammatory and antidiabetic **[148]**and neurosedative and muscle relaxant activities **[149]
			Sedative* (leaves)	In natura - plaster	

38-Gervão (MG1)^□^	*Stachytarpheta cayennensis *(Rich.) Vahl (Verbenaceae) *Garcia 054*	Native - Brazilian territory (S)	Laxative (aerial parts)	Infusion or decoction - ingestion	**Anti-inflammatory and anti-ulcerogenic properties **[150]**and hypoglycaemic constituents **[151]

39-Goiaba (SE1)^□^	*Psidium guajava *L. (Myrtaceae) *Garcia 058*	Native - Mexico to Brazil (S)	Heartburn (leaves)	Infusion or in natura - ingestion	**Antibacterial activity **[152-154]**and hepatoprotective activity **[155]
			Diarrhea (fruit)	In natura - ingestion	

40-Guaco (PE1^□^, PE2^□^, SE1^Δ^)	*Mikania glomerata *Spreng. (Asteraceae) *Garcia 032*	Native - northeast to southeast Brazil (S)	Bronchitis* (leaves)	Syrup - ingestion	**Analgesic and anti-inflammatory activities **[156], **bronchodilator activity **[157]**and antiophidian properties **[158]

41-Guanxuma (SE1)^Δ^	*Sida rhombifolia *L. (Malvaceae) *Garcia 067*	Exotic - Brazilian territory (S)	Sedative (aerial parts)	Infusion - ingestion or inhalation	**Cytotoxicity, antibacterial activity **[159]**and antioxidant **[160]

42-Guiné (SE1)^Δ^	*Petiveria alliaceae *L. (Phytolaccaceae) *Garcia 004*	Native - north Brazil (S)	Sedative (aerial parts)	Environment purifier - inhalation	**Antimicrobial substance **[161], **antimitotic action **[162], **anti-inflammatory and analgesic effects **[163], **antibacterial and antifungal activity **[164]**and antioxidant **[165]
			Muscle pain* (leaves)	Decoction - massage	

43-Hortelã (MG1, PE1)^□^	*Mentha arvensis *L. (Lamiaceae) *Garcia 031*	Exotic - Brazilian territory (C)	Bronchitis* (leaves)	Syrup - ingestion	**Antifungal property **[166], **vasodilatory actions **[167], **antioxidative activity **[168], **antibacterial properties **[107]**and insect repellents and fumigants **[109]
			Laxative (leaves)	Infusion - ingestion	

44-Hortelã-grande (PE1)^□^	*Plectranthus amboinicus *(Lour.) Spreng. (Lamiaceae) *Garcia 073*	Exotic - Brazilian territory (C)	For digestion and urine with blood (leaves)	Infusion - ingestion	**Scorpion venon antidote **[169]**and antimicrobial activity **[92]
			Cough (leaves)	Syrup - ingestion	

45-Impatiens (PE1)^Δ^	*Impatiens hawkeri *W. Bull. (Balsaminaceae) *Garcia 044*	Exotic - Brazilian territory (C)	Toxic (whole plant)	In closed environment causes tearing, allergy and headache	No data found

46-Jarnaúba (PE1)^Δ^	*Synadenium grantii *Hook. F. (Euphorbiaceae) *Garcia 074*	Exotic - southeast to northeast Brazil (C)	Toxic (whole plant)	Restricted use	**Healing action and anti-hemorrhagic **[170]
			Stomach cancer (latex)	Macerate - ingestion	

47-Jurubeba (MG1, SE1, PE2)^□^	*Solanum variabile *Mart. (Solanaceae) *Garcia 056*	Native - southeast and south Brazil (S)	Sedative (leaves)	Infusion - ingestion	**Antiulcerogenic activity **[171]
			Laxative (powder fruit)	In natura - ingestion	

48-Limão (MG1)^□^	*Citrus aurantifolia *(Christm.) Swingle (Rutaceae) *Garcia 063*	Exotic - Brazilian territory (C)	Fever (leaves)	Infusion - ingestion	**Mosquito repellent activity **[172]

49-Losna (SP1, SE1, PE2)^□^	*Artemisia absinthium *L. (Asteraceae) *Garcia 049*	Exotic - Brazilian territory (S)	Laxative (aerial parts)	Infusion - ingestion	**Acaricidal properties **[173], **antifungal and antibacterial **[174]**and antioxidant activities **[175]

50-Malva-branca (SE1)^□^	*Waltheria indica *L. (Sterculiaceae) *Garcia 077*	Native - Brazilian territory (S)	Gingivitis* (leaves)	Infusion - gargling	**Anti-inflammatory activities **[176]
			Inflammation in the mouth and/or throat* (leaves)		

51-Malva-de-cheiro (MG1)^□^	*Malva sylvestris *L. (Malvaceae) *Garcia 059*	Exotic - south and southeast Brazil (S)	Wounds in the body (roots)	Medicinal wine - ingestion	**Skin anti-aging property **[177]

52-Mamão-papaia (PE1)^□^	*Carica papaya *L. (Caricaceae) *Garcia 062*	Exotic - Brazilian territory (C)	Bronchitis* (powder fruit)	Syrup - ingestion	**Abortive **[178], **antibacterial activity **[179], **diuretic **[180]**and healing and abortive effects **[181]

53-Mandioca (SE1)^□^	*Manihot esculenta *Crantz (Euphorbiaceae) *Garcia 050*	Native - Brazilian territory (C)	conjunctivitis/sty* (dew on the leaves)	In natura - topic	**Analgesics and anti-inflammatory effects **[182]

54-Manjericão (MG1)^□^	*Ocimum basilicum *L. (Lamiaceae) *Garcia 061*	Exotic - Brazilian territory (C)	Bronchitis* (leaves)	Syrup - ingestion	**Antibacterial **[183], **mosquito repellent activity **[184], **antimicrobial activity **[185], **antigiardial activity **[186]**and decreases cholesterol **[187]

55-Maravilha (SP1, PE2)^□^	*Mirabilis jalapa *L. (Nyctaginaceae) *Garcia 065*	Native - Brazilian territory (C)	Healing wounds* (aerial parts)	Infusion - plaster	**Antibacterial effect **[188]**and antimicrobial **[189]

56-Maria-pretinha (MG1)^□^	*Solanum americanum *L. (Solanaceae) *Garcia 070*	Native - Americas (S)	Sore throat* (aerial parts)	Infusion - gargle	**Treatment of protozoal infections (*American trypanosomes*) **[190]**and moderate antioxidant activity **[191]

57-Mentrasto (PE1)^□^	*Ageratum conyzoides *L. (Asteraceae) *Garcia 010*	Native - southeast to northeast Brazil (S)	Bronchitis* (leaves)	Infusion - ingestion	**Anti-inflammatory **[192], **toxic **[193], **antibacterial **[194]**and insecticidal activity **[195]
			Rheumatism* (whole plant)	Infusion - bath	

58-Mentruz, erva-de-santa-maria (PE1^#^, SE1^□^)	*Chenopodium ambrosioides *L. (Chenopodiaceae) *Garcia 006*	Native - south and southeast Brazil (S)	Muscle pain (aerial parts)	Decoction - massage	**Insecticidal properties **[196], **antifungal, antiaflatoxigenic and antioxidant activity **[197]**and mosquito repellent activity **[71]
			Lesions in bone (aerial parts)	In natura - plaster	
			Worm* (aerial parts)	Infusion - ingestion	
			Bronchitis (aerial parts)	Syrup - ingestion	

59-Milho (SE1)^□^	*Zea mays *L. (Poaceae) *Garcia 023*	Exotic - Brazilian territory (C)	Bronchitis (flowers)	Syrup - ingestion	No data found
			Blood purifier and diuretic (flowers)	Infusion - ingestion	

60-Novalgina (MG1, SE1)^□^	*Achillea millefolium *L. (Asteraceae) *Garcia 015*	Exotic - south and southeast Brazil (C)	Sedative (leaves)	In natura - ingestion	**Antioxidant and antimicrobial activity **[198]

61-Pariparoba (MG1)^□^	*Piper umbellatum *L. (Piperaceae) *Garcia 072*	Native - Tropical America (S)	Belly ache and liver pain (leaves)	Infusion - ingestion	**Antioxidant **[199]**and antifungal activity **[200]

62-Picão (MG1)^□^	*Calea *sp. (Asteraceae) *Garcia 036*	No data (S)	Diuretic (leaves)	Infusion - ingestion	Not consulted

63-Picão-preto (MG1, PE1)^□^, Picão-branco (SP1)^□^	*Bidens pilosa *L. (Asteraceae) *Garcia 020*	Native - tropical America (S)	Blood purifier (whole plant)	Infusion - ingestion	**Hypotensive effects **[201], **anti-inflammatory activity **[202], **anticancer and antipyretic activity **[203], **antimicrobial **[204]**and antitumor potential **[205]
			Healing wounds* (whole plant)	In natura - plaster	
			Wounds in the body* (roots)	Medicinal wine - ingestion	

64-Pinhão-roxo (SP1)^□^	*Jatropha gossypiifolia *L. (Euphorbiaceae) *Garcia 017*	Native - southeast to northeast Brazil (S)	Laxative (powder fruit)	In natura - ingestion	**Antimalarial effects **[206], **hypotensive and vasorelaxant effects **[207]

65-Poejo (MG1, PE2)^□^	*Mentha pulegium *L. (Lamiaceae) *Garcia 029*	Exotic - Brazilian territory (C)	Bronchitis (leaves)	Syrup - ingestion	**Larvicidal activity **[208], **acaricidal effects **[209]**and insecticidal properties **[210]

66-Pucunã (SE1)^□^	*Fevillea passiflora *Vell. (Cucurbitaceae) *Garcia 022*	Native - North and southeast Brazil (S)	Toxic - abortive (seeds)	In natura - ingestion	No data found

67-Quebra-pedra (SP1, PE1, PE2, SE1)^□^	*Phyllanthus caroliniensis *Walter (Euphorbiaceae) *Garcia 024*	Native - USA to Brazil (S)	Kidney stone* (aerial parts)	Infusion or decoction - ingestion	**Antinociceptive action **[211]

68-Quitoco (SE1)^□^	*Pluchea sagittalis *(Lam.) Cabrera (Asteraceae) *Garcia 042*	Native - south and southeast Brazil (S)	Diuretic (aerial parts)	Infusion - ingestion	**Anti-inflammatory activity **[212]

69-Rubim (MG1, SP1)^□^	*Leonurus sibiricus *L. (Lamiaceae) *Garcia 002*	Exotic - Brazilian territory (C)	Healing wounds* (aerial parts)	In natura - plaster	**Stimulating action on the uterus **[213], **analgesic and anti-inflammatory activity **[214]**and antibacterial activity **[215]

70-Sabugueiro (MG1)^□^	*Sambucus canadensis *L. (Caprifoliaceae) *Garcia 025*	Native - Brazilian territory (S)	Bronchitis* (flowers)	Syrup - ingestion	**Infectious diseases and antioxidant activity **[216]

71-Salsa-parreira (SE1)^□^	*Jacaranda *sp. (Bignoniaceae) *Garcia 011*	No data (S)	External allergies, wounds in the body and purifier (leaves)	Decoction - bath	Not consulted

72-Samba-caitá (SE1)^□^	*Hyptis *sp. (Lamiaceae) *Garcia 041*	No data (S)	Belly ache (leaves)	In natura - ingestion	Not consulted

73-Serralha (PE1)^□^	*Sonchus oleraceus *L. (Asteraceae) *Garcia 016*	Exotic - Brazilian territory (S)	Diabetes (leaves)	In natura - ingestion	**Larvicidal potential **[217]

74-Sete-sangria (MG1^□^, SP1^□^, SE1^Δ^)	*Cuphea carthagenensis *(Jacq.) J. F. Macbr. (Lythraceae) *Garcia 007*	Native - Brazilian territory (S)	Intestinal infections and heart problems* (aerial parts)	Infusion - ingestion	**Antiinflammatory and antinociceptive activities **[218], **vasorelaxant properties **[219], **treat high levels of cholesterol and triglycerides **[220]

75-Sofre-do-rim-quem-qué (MG1)^#^	*Cissus *sp. (Vitaceae) *Garcia 053*	No data (S)	Kidney stone (leaves)	Infusion - ingestion	Not consulted

76-Tanchagem (SP1, PE2)^□^	*Plantago *sp. (Plantaginaceae) *Garcia 008*	No data (S)	Anti-inflammatory - mouth and throat (leaves)	Decoction - gargling	Not consulted

77-Vassourinha (SE1, PE2)^□^	*Scoparia dulcis *L. (Scrophulariaceae) *Garcia 014*	Native - Brazilian territory (S)	Hip pain/kidneys (leaves)	Decoction - bath	**Antitumor-promoting activity **[221], **antioxidant **[222], **antimicrobial and antifungal activities **[223]

78-Velando (SE1)^□^	*Croton fuscescens *Spreng (Euphorbiaceae) *Garcia 013*	Native - Brazilian territory (S)	Inhibits the growth of skin stains/wounds in the body (resin)	In natura - topic	No data found

* their popular and scientific names, geographical origin and distribution, if cultivated or spontaneous, uses, parts utilized, formula, route of administration and pharmacological studies. Marked by (^□^) the 68 plants whose use had been maintained by the respective migrant, while 14, marked by (^Δ^) are those whose applications have been incorporated by migrants, finally, 3 (^#^) are replacements. The matches between the uses proclaimed by the interviewees and pharmacological data have been posted by (*).

From the 65 species identified, it was observed that 33 are native to Brazil while the other 32 are exotic, demonstrating the great floral diversity of the region, which was influenced by European and African people during the civilizing process in Brazil. Furthermore, of the 78 specimens recorded, 54% (42) are spontaneous or were already available in Diadema when interviewees arrived there, while 46% (36) were grown by the migrants, acquired in free markets, or brought from other regions of the country during migration. Below, we describe the four 'dynamics of use' categories observed during this study.

#### Maintenance of use

According to the interviewees, 68 of the 78 specimens cited in the present study, were used in their homelands (highlighted with □ in Table 2). The maintenance of their uses was possible since most of them were available in Diadema, though some were brought from their homelands. SE1 brought four plants from Aquidabã - Sergipe state, for pain relief because they are not available or are more potent than the ones found in Diadema: "bálsamo" (*Sedum *sp.), "anador" (*Alternanthera *sp.), "eucalipto/vick" (*Eucalyptus globulus *Labill.) and "novalgina" (*Achillea millefolium *L.).

#### Incorporation of use

Fourteen of the 78 specimens listed in Table 2 came to be used by migrants when they arrived in Diadema (highlighted with Δ in Table 2). These incorporations occurred in several ways: through information given by neighbours; through local media, e.g., television, radio, magazines; or through personal efforts, guided by plant organoleptic properties or even by the theory of signatures. This theory, formulated by Paracelsus (XVI century), assumes that characteristics and virtues of herbs can be recognised by their external appearance or "signature" (picture, shape, colour). Finally, observing the relationship between animals and plants can be a valuable guide. PE1 noted that dogs consume "sete-sangria" (*Cuphea carthagenensis *(Jacq.) J. F. Macbr.) when they have diarrhoea; and because it seemed to alleviate their symptoms, he started to use this plant for the same purpose.

The migrants incorporated several plants after their arrival in Diadema to treat typical diseases of larger cities: "cipó-cruz" (*Serjania *sp.) to combat high cholesterol; and "guanxuma" (*Sida rhombifolia *L.) and "guiné" (*Petiveria alliaceae *L.) for anxiety. Also included in this category was knowledge concerning local toxic plants, e.g., alamanda-amarela (*Allamanda cathartica *L.) and azaléia (*Rhododendron simsii *Planch.), detailing the risks associated with their consumption.

#### Replacement of use

Three plants used by migrants in their cities of origin were replaced because they were not available or were less effective than plants present in Diadema (highlighted with # in Table 2). Most of these replacements were made according to the criteria listed in the previous section.

The interviewee MG1 explained that in his homelands, he used "quebra-pedra"* (*Phyllanthus *cf. *caroliniensis *Walter - Euphorbiaceae) for kidney stone disturbance, but when he arrived in Diadema, he found another plant, "sofre-do-rim-quem-qué" (*Cissus *sp.), that seemed to have a stronger effect.

Another interviewee, PE1, reported that the bark and seeds of "amburana-de-cheiro"* (*Amburana *cf. *cearensis *(Allemão) A.C. Sm. - Fabaceae s.l.) were widely used for anti-inflammatory therapy in Pernambuco state but had to be replaced by "mentruz" (*Chenopodium ambrosioides *L.) because the former was not found in Diadema. In addition, SE1 had to replace "pau-de-sapo"* (*Pouteria *cf. *melinoniana *Boehni - Sapotaceae), whose leaves were used for chronic wounds, with "carrapicho" (*Acanthospermum australe *(Loefl.) Kuntze).

The vernacular names of some plants are registered trademarks of allopathic medicines and active ingredients, e.g., Novalgina^® ^(*Achillea millefolium*) and Vick^® ^(*Eucalyptus globulus*) for sinusitis, and Anador^® ^(*Alternanthera *sp.), which is used as a sedative and for general pain. Contact between migrants and allopathic medicine thus led to the 'baptisms' of these plants, following the observation that both, the commercially available products and herbal source have similar effects, as reported by Pires [43].

#### Discontinued use

According to MG1, the following plants used in his homeland fell into disuse because they were not found in Diadema, although he tried to acquire them from local commercial sources: "quina"* (*Strychnos *cf. *pseudoquina *A. St. Hil - Loganiaceae), whose root is used to combat pain in the stomach and intestine; bark oil of "jatobá"* (*Hymenaea *cf. *courbaril *L. - Fabaceae s.l.), used for combat wounds; "batata-de-purga"* (*Operculina *cf. *macrocarpa *(L.) Urb - Convolvulaceae), whose tuber is ingested as a purgative and to clean the blood; bark and leaf of "jalapa"* (*Mirabilis *cf *jalapa *L. - Nyctaginaceae), used to clean the blood; tea of "junco"* (*Cyperus *cf. *esculentus *L. - Cyperaceae), whose root is used for inflammation; bark or seed of "emburana"* (*Amburana *cf. *cearensis *- Fabaceae s.l.), used for migraine and sleeping; and bark of "angico"* (*Anadenanthera *cf. *colubrina *(Vell.) Brenan - Fabaceae s.l.), prepared as a tea for pain in the body and fever. These plants were not described in Table 2, since they could not be collected and identified as well.

#### Plants used for therapeutic purposes

Of the 78 plants, 10 carry some restrictions, as they can be toxic depending on the dose, route or part utilised (Table 2). The uses described in Table 2 are written just as they were reported by the interviewees. The 68 plants used exclusively for medicinal purposes were cited for 41 complaints, which were grouped into 12 functional categories according to bodily system, as detailed in Table 3. Thus, gastrointestinal disturbances include the following complaints (numbers of medicinal plants reported): endoparasitosis (1), ulcer (1), diarrhoea (1), bellyache (2), heartburn (1), intestinal infections (1), liver pain (3). This category also includes plants used to improve digestion (1), to treat tables of haemorrhoid (1), as laxatives (10) and to purify the stomach (2), comprising a total of 24 plants employed in 44 formulas.

**Table 3:** The 12 categories of use comprising the 41 complaints, their total and partial number of plants cited by the five migrants.

Category of use	Complaints (number of plants cited)	Total number of plants
**1- *Gastrointestinal disturbances***	To combat worms (1), ulcer (1), diarrhoea (1), bellyache (2), heartburn (1), intestinal infections (1), liver pain (3), to improve digestion (1), hemorrhoid (1), as laxative (10) and for stomach purify (2)	24

**2- *Inflammatory processes***	As anti-inflammatory (3) and healing (6), to treat sty/conjunctivitis (2), inflammation in the mouth/throat (3), rheumatism (2), sinusitis (2) and gingivitis (1)	19

**3- *Respiratory problems***	To combat cough (1), bronchitis (15) and as expectorant (2)	18

**4- *Anxiolytic/hypnotics***	As sedative (11)	11

**5-O*steomuscular problems***	To ease back pain (1), muscles pain (6), hip pain (1), prevent osteoporosis (1) and to treat lesions in bone (1)	10

**6- *Dermatological problems***	To combat external allergies (2), wounds in the body (5) and inhibits the growth of skin stains (1)	8

**7- *Genitourinary disturbances***	As diuretic (5), to combat kidney stone (2) and treating urine with blood (1)	8

**8- *Endocrine system***	To reduce cholesterol (1) and diabetes (3)	4

**9- *Cardiovascular problems***	Treat heart problems (1) and as blood purifier (2)	3

**10- *Immunological problems***	To combat breast cancer (1) and stomach cancer (1)	2

**11- *Analgesics***	Earache (2)	2

**12- *Fever***	To combat fever (1)	1

***Total***		110*

* Some plants have been cited for more than one complaint, so the total number of plants above (110) is higher than the ones indicated by the interviewees.

The most relevant categories of use, measured by number of species employed, were gastrointestinal disturbances (30.8% of plants), inflammatory processes (24.4%) and respiratory problems (23.1%). As seen in Table 4, the group of illnesses representing immunological problems obtained the highest informant consensus factor value (*Fic *= 0.66), while the other categories presented *Fic *values lower than 0.5. These low values reflect the diversity of knowledge displayed by migrants, which can probably be attributed to different cultural influences during their migrations through Brazilian territory. Furthermore, the small number of interviewees may have resulted in low values of *Fic*.

**Table 4:** Values of Informant consensus factor (*Fic*) for each category of use, considering the plants cited by the five Diadema's migrants.

SN	Category of use	Plant specimen	% All Species	Use citation	% All use citation	*F*ic
1	**Gastrointestinal disturbances**	24	30.77	44	25.29	0.46

2	**Inflammatory processes**	19	24.36	28	16.09	0.33

3	**Respiratory problems**	18	23.07	31	17.82	0.43

4	**Anxiolytic/hypnotics**	11	14.10	19	10.92	0.44

5	**Osteomuscular problems**	10	12.82	13	7.47	0.25

6	**Dermatological problems**	8	10.26	11	6.32	0.3

7	**Genitourinary disturbances**	8	10.26	13	7.47	0.41

8	**Endocrine system**	4	5.13	5	2.87	0.25

9	**Immunological problems**	2	2.56	4	2.30	0.66

10	**Cardiovascular problems**	3	3.84	3	1.72	0

11	**Analgesics**	2	2.56	2	1.15	0

12	**Fever**	1	1.28	1	0.57	0

The parts of the plants most often used in the formulas were leaves (45.4%) and other aerial parts (22.7%). The most common formula was the infusion (37.8%), followed by in natura (17.6%) and syrup (10.1%). The most cited route of administration was ingestion (51.3%), followed by inhalation (8.4%) and topical (3.4%).

#### Plants with restrictions on use and/or toxic

Among the 10 specimens with restrictions on use, 6 were designated as only toxic: "alamanda-amarela" (*Allamanda cathartica*), "algodão-do-mato" (*Asclepias curassavica *L.), "amendoim-bravo/burra-leiteira" (*Euphorbia heterophylla *L.), "azaléa" (*Rhododendron simsii*), "comigo-ninguém-pode" (*Dieffenbachia *sp.) and "impatiens" (*Impatiens hawkeri*). The interviewees explained that depending on the dose, the latex of "alamanda-amarela" and "amendoim-bravo" can cause discomfort or even blindness. According to Oliveira et al. [44], the leaves of *Dieffenbachia picta *Schott contain calcium oxalate, which damages the oral mucosa and provokes pain and oedema, while the leaves of *Allamanda cathartica *contain cardiotonic glycosides and induce intense gastrointestinal disturbances.

Although reported as toxic, the latex of two other plants can be used at low doses to treat breast and stomach cancer: "avelóz" (*Euphorbia tirucalli *L.) and "jarnaúba" (*Synadenium grantii *Hook. F.), respectively. The sap of "embaúba" (*Cecropia pachystachya *Tréc.) was indicated as toxic, but its fruits are used to combat bronchitis. Finally, the seeds of "pucunã" (*Fevillea passiflora *Vell.) are toxic, being indicated as abortive. In a recent study, Rodrigues [45] also described plants with restrictions of use as reported by three Brazilian cultures: the Krahô Indians use two plants as abortives in a single prescription: "aprytytti" (*Acosmium dasycarpum *(Vogel) Yakovlev) and "ahkryt" (*Anacardium occidentale *L.) (Anacardiaceae); their barks are boiled, and the beverage is ingested in at dawn. It is an extremely bitter beverage, rich in tannin and therefore extremely astringent.

#### Pharmacological data

As can be seen in Table 2, 57 species (73.1%) were featured in previous pharmacological studies. For 30 of these species (52.6%), the uses cited by the migrants showed some similarity to the investigated effects/actions, demonstrating concordance between popular knowledge and academic science (marked with an asterisk in Table 2).

**Table 5:** The 6 categories of use comprising the 9 complaints, their respective number of animals mentioned by the migrant PE2.

Category of use	Complaints (number of animals)
**1-Respiratory problems**	bronchitis (7), asthma (4)

**2-Central nervous system**	epilepsy (1)

**3-Inflammatory processes**	rheumatism (1)

**4-Dermatological problems**	wounds in the body (1), skin cancer (1)

**5-Analgesics**	back pain (1)

**6-Cardiovascular problems**	treat heart problems (1), hemorrhage (1)

**Total**	18*

* some animals have been cited for more than one complaint, so their total number above (18) is higher than the number of animals indicated: 12.

#### Animals used for therapeutic purposes and dynamics of use

From the five interviewees, only one (PE2) offered knowledge on the medicinal uses of 12 animals. They belong to four taxonomic classes: Reptilia (6 species), Insects (3), Mammalia (2) and Amphibia (1). However, the interviewee has used only two animals since he arrived in Diadema, the other ten animals fell into disuse because they are not available in this city. The two animals were collected, identified and deposited in the Museum of Zoology-USP: ant (*Atta sexdens *L.) and cockroach (*Periplaneta americana *L.). These species belong to the *maintenance of use *category (highlighted with □ in Table 1). The other ten species therefore belong to the *discontinued use *category (highlighted with ^Ο ^in Table 1) which could not be collected. Their identifications were made by PE2 through consulting images from books (as described in **Methodology**). For three animals (snake, alligator and giant water bug) PE2 could only hesitantly confirm their identity, probably due to the great diversity of these animals in Brazil. Therefore, they are denoted in Table 1 as probably belonging to one of three possible genera.

The animals were used in 14 different medicinal formulas, with the skin most commonly used (33.3%), followed by whole animal (20.0%), bone (13.4%), fat (6.7%), rattle (6.7%), tooth (6.7%), anthill (6.7%) and turtleshell (6.7%). Some studies conducted in Brazil show that concomitant data corroborate and sustain these uses [46-50]. The formulas were cited for the treatment of nine complaints, which were grouped into six functional categories, as shown in Table 5. The most commonly cited formula was powder (66.7%), followed by in natura (20%). The most frequent route of administration was ingestion (78.6%).

The most common complaint involved respiratory problems (58.4%; 7 animals) followed by central nervous system (8.3%), inflammatory processes (8.3%), dermatological problems (8.3%), analgesics (8.3%), cardiovascular problems (8.3%) as shown in Table 5. The high humidity of the region (with annual rainfall between 1.000 and 1750 mm) [21] is known to lead to bronchitis, cough and asthma. This may explain why so many plants and animals were used to treat respiratory disturbances in Diadema, which has been shown in studies of the Sistema Único de Saúde [51] to be the second largest cause of death in Diadema - 14,4%.

Many animals have been used for medical purposes since antiquity [52-55]. Despite the existence of several ethnopharmacological studies suggesting the bioactive potential of Brazilian fauna [37,56-61], only marine animals have been investigated by chemical and pharmacological methods [62-64]. No pharmacological data was found in the literature for the five animals identified in the present study: rattlesnake (*Crotalus *cf. *durissus *L.), capybara (*Hydrochoerus *cf. *hydrochaeris *L.), iguana (*Iguana *cf. *iguana *L.), ant (*Atta sexdens*) and cockroach (*Periplaneta americana*). The lack of information available on medicinal animal products leads us to conclude that this is a largely unexplored topic in Brazil and that future pharmacological studies should confirm the potential therapeutic value of these species.

## Conclusion

The migrant interviewees demonstrated knowledge about the medicinal and toxic properties of plants and animals available in the Atlantic Forest remnants of the municipality of Diadema. Migration contributed to the expansion of knowledge regarding the use of natural resources, especially through the processes of resource replacement and/or incorporation. Moreover, the maintenance of original uses of certain resources demonstrates their value in the migrants' therapeutic practices.

The seven plants [*Impatiens hawkeri *W. Bull., *Artemisia canphorata *Vill., *Equisetum arvensis *L., *Senna pendula *(Humb. & Bonpl. ex Willd.) H.S. Irwin & Barneby, *Zea mays *L., *Fevillea passiflora *Vell. and *Croton fuscescens *Spreng)] and the two animals (*Atta sexdens *and *Periplaneta americana*) that showed maintenance of use among migrants during their displacement in Brazilian territory, have not been studied by pharmacologists yet. These species should be highlighted in further investigations because the maintenance of use during human migrations can be indicative of bioactive potential.

This work also demonstrates the impossibility of sharing benefits related to property rights with cultures under certain circumstances, as the dynamic use of natural resources presents particularly varied influences. The interviewed migrants had passed through several Brazilian cities and were exposed to distinct vegetation and cultures. In this migration, they have passed on and incorporated knowledge in an intensive exchange where formulas and uses are mixed and re-invented as a result of contact between cultures.

## Competing interests

The authors declare that they have no competing interests.

## Authors' contributions

Author DG performed the fieldwork. Author MVD identified the animal specimens. Author ER supervised the research works. All authors drafted, wrote, read and approved the final manuscript.
